# Genome-Wide Identification and Analysis of Carbohydrate-Binding Modules in *Colletotrichum graminicola*

**DOI:** 10.3390/ijms26030919

**Published:** 2025-01-22

**Authors:** Ya-Fei Wang, Qiu-Li Huang, Xin-Yu Chen, Hong-Lian Li, Jia-Xin Chang, Yu Zhang, Yi-Wen Wang, Yan Shi

**Affiliations:** 1 College of Plant Protection, Henan Agricultural University, Zhengzhou 450002, China; 15729397930@163.com (Q.-L.H.); honglianli@henau.edu.cn (H.-L.L.); changjiaxin2104@163.com (J.-X.C.); 18436618796@163.com (Y.Z.); 15136463855@163.com (Y.-W.W.); 2 State Key Laboratory for Biology of Plant Diseases and Insect Pests, Institute of Plant Protection, Chinese Academy of Agricultural Sciences, Beijing 100193, China; 821012410333@caas.cn

**Keywords:** *Colletotrichum graminicola*, carbohydrate-binding modules, gene family, expression pattern

## Abstract

*Colletotrichum graminicola* is the causative agent of both maize stem rot and leaf blight, which are among the most damaging diseases affecting maize. Carbohydrate-binding modules (CBMs) are protein domains that lack catalytic activity and are commonly found alongside carbohydrate-hydrolyzing enzymes in fungi. A comprehensive examination of the *C. graminicola* TZ-3 genome resulted in the identification of 83 *C. graminicola CBM* (*CgCBM*) genes, which are characterized by distinct gene structures and protein motifs. Subcellular localization analysis revealed that the majority of CgCBM proteins were localized in the extracellular space. Investigation of the promoter regions of *CgCBM* genes uncovered a variety of responsive elements associated with plant hormones, including abscisic acid and methyl jasmonate response elements, as well as various stress-related response elements for drought, cold, defense, and other stress factors. Gene ontology analysis identified the primary functions of *CgCBM* genes as being linked to polysaccharide metabolism processes. Furthermore, the 83 *CgCBM* genes exhibited varying responses at different time points during *C. graminicola* infection, indicating their contribution to the fungus–maize interaction and their potential roles in the fungal pathogenic process. This study provides essential insights into *CgCBMs*, establishing a crucial foundation for further exploration of their functions in the mechanisms of fungal pathogenicity.

## 1. Introduction

The cell wall serves as the primary defense barrier against pathogen intrusion. Numerous pathogens secrete substantial amounts of cell wall-degrading enzymes (CWDEs) to accelerate the decomposition of cell walls, and these enzymes are critical virulence factors [1,2,3]. Specific CWDEs, such as endo-α-1,4-galacturonase, found in fungi, function as pathogen-associated molecular patterns (PAMPs) that trigger pattern-triggered immunity (PTI) [4,5,6]. Within CWDEs, there are carbohydrate-binding modules (CBMs), which are non-catalytic protein domains typically associated with carbohydrate-hydrolyzing enzymes in fungi, playing a role in activating PTI responses [7,8,9].

In general, CBMs are linked to the catalytic domains through peptide linkers, although a few CBMs exist independently. They facilitate hydrolysis by increasing the proximity between the enzymes and the substrates, which help to position the catalytic domain attached to the CBMs at the surface of the substrates. This positioning plays a crucial role in the enzymatic conversion of cellulose and various polysaccharides [10,11]. Additionally, CBMs help maintain their conformation, thereby enhancing their thermal stability [12]. Some CBMs, which exhibit increased binding affinity, can also improve the processing efficiency of the enzyme [13]. Furthermore, CBMs influence the substrate specificity of catalytic domains by modifying the binding mode of the enzymes [14]. It is known that certain CBMs possess side chains that target polysaccharides and specifically promote their hydrolysis, making CBMs a highly attractive research focus for enhancing cellulose hydrolysis [15,16].

CBMs can also be purified as individual proteins [17,18,19]. In carbohydrate-active enzymes (CAZymes), such as glycoside hydrolases (GHs), CBMs are typically located at either the N-terminus or C-terminus of these proteins [20,21]. They can also be found between two catalytic domains [22]. These domains may exhibit high conservation or significant homology [23,24]. However, in certain cases, tandem CBMs may consist of modules with distinct substrate specificities. For instance, the gas vesicle membrane α-sialidase from *Clostridium perfringens* possesses two CBM-ones that bind to galactose and the other that specifically targets sialic acid [25].

*C. graminicola* is a semi-biotrophic fungus responsible for causing stem rot and leaf blight in maize (*Zea mays* L.), which is commonly found in cornfields [26]. These diseases result in a significant reduction in maize yield and can affect various maize hybrids [27,28,29]. The conidia present on maize residues serve as the primary source of inoculation for this disease in the field. Conidiospores, the predominant type of conidia, are dispersed to maize seedlings through splashing rainwater [27]. Upon contact with the host cell wall, the spores germinate, forming appressoria at the tip of the germ tube. These appressoria produce penetration pegs that breach the host cell wall, allowing the fungi to invade the cells [30,31,32,33].

The establishment of a high-quality genome sequence for *C. graminicola* has not only paved the way for molecular biology investigations but also facilitated the analysis of genes associated with pathogenic mechanisms [34]. This study identified all *CBM* family genes in the genome of *C. graminicola* TZ-3 and subjected them to a comprehensive analysis. Phylogenetic analysis was employed to elucidate the interrelationships among these genes, while online tools were utilized to predict their characteristics. Furthermore, a differential expression analysis of *CBM* genes during the infection of maize hosts by *C. graminicola* revealed a potentially significant role for these genes in the pathogenic mechanism. Overall, this research has enhanced our understanding of *C. graminicola CBM* (*CgCBM*) genes and established a foundational knowledge base for future in-depth investigations into their involvement in pathogenic mechanisms.

## 2. Results

### 2.1. Physicochemical Analysis of CgCBM Genes

A total of 83 *CBM* family genes were identified in the genome of *C. graminicola* TZ-3, numbered as *CgCBM1*-*CgCBM83*. Their comprehensive characteristics were presented in Table 1. All CgCBM proteins are categorized into 16 distinct superfamilies. Notably, 19 CgCBM proteins are classified within the CBM1 superfamily, 16 within the CBM50 superfamily, and 15 within the CBM18 superfamily. The CgCBM proteins varied from 80 to 1778 amino acids (aa), and the predicted molecular weights were between 8.18 and 198.30 kDa. Most of the CgCBM proteins are acidic proteins (PI value < 6.5). The average hydropathy scores were between −1.097 and −0.13, indicating the hydrophilic nature of the CgCBM proteins. Additionally, subcellular localization revealed that 68 CgCBM proteins were extracellularly localized, 7 CgCBM proteins were located in the cytoplasm, 4 CgCBM proteins were located in the mitochondria, 3 CgCBM proteins were located in the nucleus, and CgCBM52 was located on the plasma membrane (Table 1).

### 2.2. Phylogenetic Relationships

**Figure 1:**
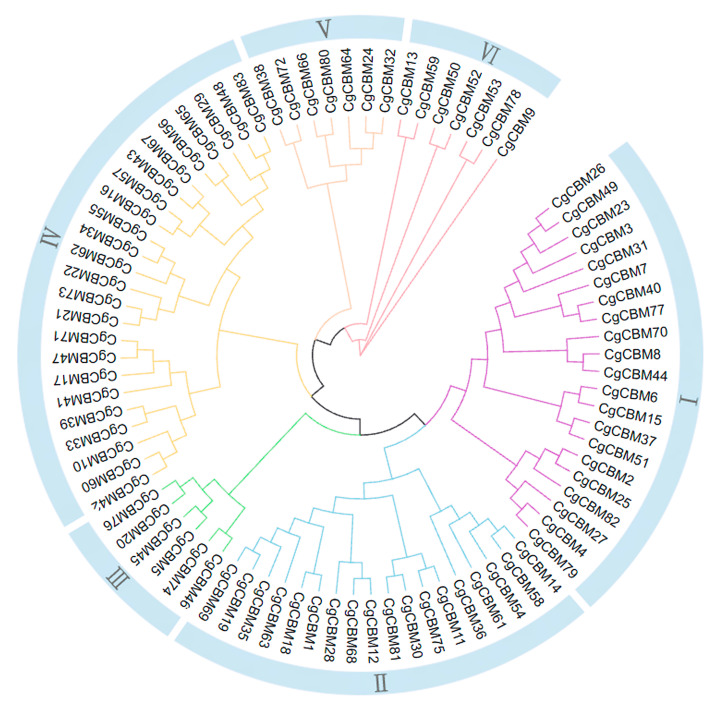
Neighbor-joining phylogenetic tree of carbohydrate-binding module (CBM) proteins from *Colletotrichum graminicola*. Note: “Cg” represents *C. graminicola*.

In order to elucidate the phylogenetic relationships among CgCBMs, MEGA5.0 was used to construct a phylogenetic tree of the 83 CgCBM protein sequences (Figure 1, Table S1). These proteins were categorized into six groups, I, II, III, IV, V, and VI, containing 21, 18, 6, 24, 7, and 7 members, respectively (Figure 1). The proximity of Groups I and II on the phylogenetic tree suggested a strong genetic association between them. On the contrary, the distance between Group I and Group VI was significant, indicating that the genetic relationship between them was relatively weak.

### 2.3. Structural Analysis of CgCBMs

**Figure 2:**
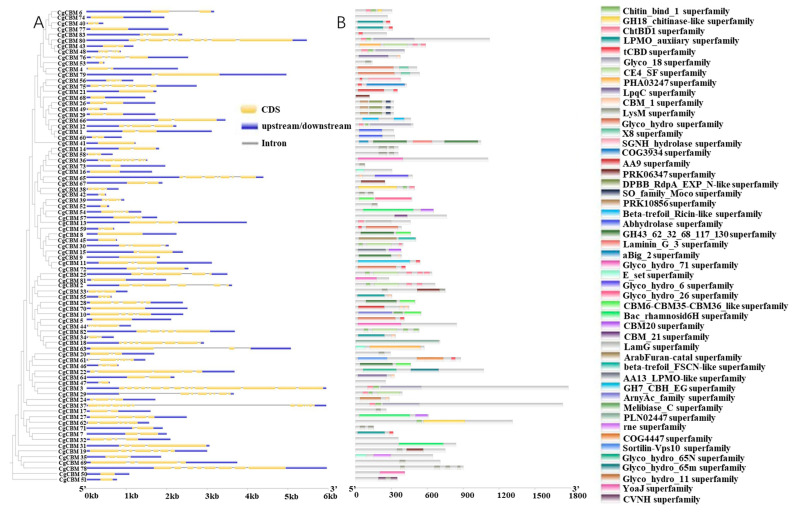
Gene structure and domain analysis of CgCBMs. Note: (**A**) Exon–intron structures of *CgCBMs*. (**B**) Domains of CgCBMs.

In order to clarify the characteristics of the *C. graminicola* TZ-3 *CBM* family, an analysis was conducted on the gene structure of the *CgCBM* family (Figure 2A, Table S1). The results showed that there were significant differences in the gene structure of the *CgCBM* family. Specifically, *CgCBM* was identified as have varying numbers of introns (ranging from 0 to 14). Notably, *CgCBM3* and *CgCBM37* had the highest number of introns, while 15 *CgCBMs* lacked introns. These results demonstrated that the gain and loss of introns took place during evolution.

A comprehensive domain analysis highlighted the diverse structural domains present in CgCBMs, where the majority of them exhibited multiple domains (Figure 2B, Table S1). For instance, CgCBM25 exhibited five domains, while CgCBM2, CgCBM41, and CgCBM43 each possessed four domains. Some CgCBMs featured dual GH and CBM domains, as exemplified by CgCBM39 with the CBM6-CBM35-CBM36_like superfamily and the Glyco_hydro_26 superfamily, and CgCBM65 harboring both the CBM_1 superfamily and the Glyco_hydro_6 superfamily.

**Figure 3:**
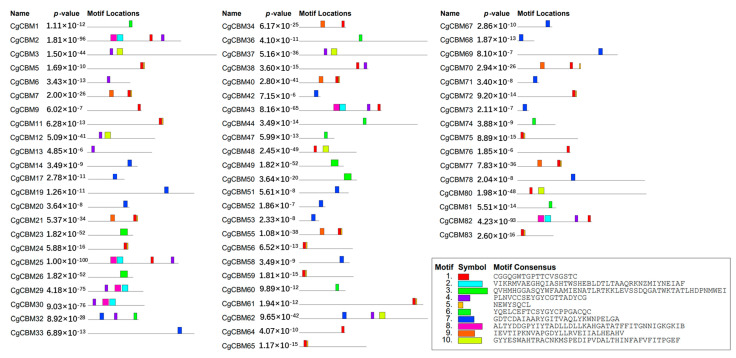
Motifs of CgCBMs predicated by MEME. Note: Distinct colored boxes denote various conserved motifs with differed sizes and sequences.

To further delve into the function of *CgCBM* genes, the Multiple Em for Motif Elicitation (MEME) website was utilized for evaluating the conserved motifs of the CgCBM proteins (Figure 3). The analysis unveiled a total of 10 motifs in the CgCBM proteins (Figure S1). Notably, motif 7 was the most common motif identified in 18 CgCBM proteins. Motif 7 was the only conserved motif in CgCBM14, CgCBM17, CgCBM19, CgCBM20, CgCBM33, CgCBM42, CgCBM51, CgCBM52, CgCBM53, CgCBM58, CgCBM67, CgCBM68, CgCBM69, CgCBM71, CgCBM73, and CgCBM78. Furthermore, CgCBM2, CgCBM25, CgCBM43, and CgCBM82 had common motifs, including motifs 2, 4, 8, and 9, with identical sequence orders observed for CgCBM2 and CgCBM25, as well as for CgCBM43 and CgCBM82. Similarly, motifs 1, 5, and 9 were shared in CgCBM7, CgCBM40, CgCBM55, CgCBM70, and CgCBM77. Consistency of motifs, such as motifs 2, 4, and 8, was observed in CgCBM29 and CgCBM30. Moreover, motifs 1 and 5 were common among CgCBM5, CgCBM11, CgCBM24, CgCBM56, CgCBM59, CgCBM61, CgCBM65, CgCBM72, and CgCBM75 (Figure 3). The similarity of the conserved motifs within the protein groups indicated the potential sharing function among these CgCBMs.

### 2.4. Protein Structure Analysis of CgCBMs

**Figure 4:**
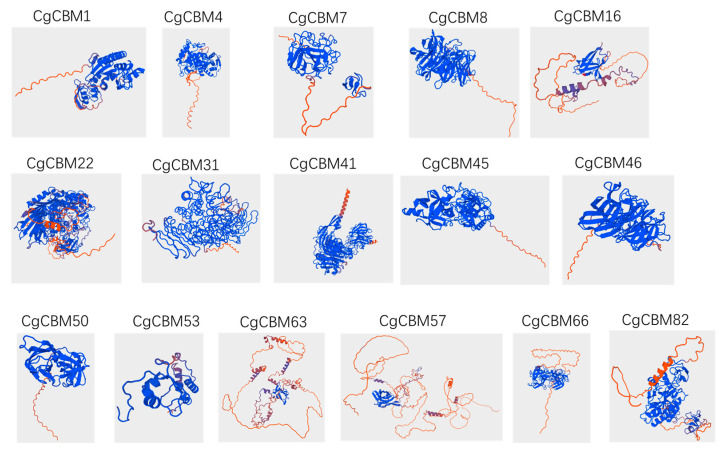
Three-dimensional models of CgCBM proteins from different superfamilies were predicted at confidence level > 0.7.

To enhance our understanding of the biological functions of CBMs in *C. graminicola*, we investigated the secondary structures of all CgCBMs, with random coils being the predominant component. Specifically, the proportions of α-helices varied from 3.91% to 38.28%, β-turns from 0 to 13.45%, random coils from 41.52% to 94.41%, and extended stands from 1.68% to 34.2% (Table S2). Furthermore, we predicted the three-dimensional (3D) protein structures of 16 CgCBMs from all different superfamilies. As illustrated in Figure 4, the 3D structures of these CgCBM proteins displayed significant differences, underscoring the structural diversity inherent in the CgCBM family.

### 2.5. Sequence Analysis of Promoter Region of CgCBM Gene Family

**Figure 5:**
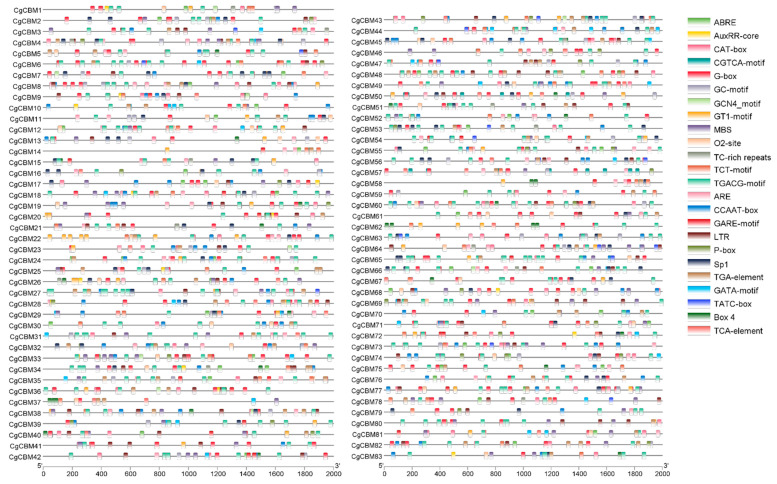
Analysis of cis-acting regulatory elements in promoter regions of *CgCBMs*. Note: Different colored boxes represent different cis-acting regulatory elements.

The promoter regions of the *CgCBM* gene family were analyzed for sequence composition using an online server to elucidate gene functions accurately. The analysis revealed the presence of various cis-acting regulatory elements (CAREs) in these regions (Figure 5, Table S3). Specifically, the promoter regions contained plant hormone response elements like MeJA (TGACG-motif and CGTCA-motif), abscisic acid (ABRE), gibberellin (P-box, GARE-motif, and TATC-box), and salicylic acid (TCA-element) response elements. They also encompassed elements related to growth and development, such as light response elements (G-Box, Sp1, TCT-motif, GATA-motif, and GT1-motif) and zein metabolic regulation response elements (O2-site). In addition, the promoter regions contained numerous response elements associated with low temperature (LTR), drought (MBS), and defense (TC-rich repeats). Notably, MeJA response elements were the most abundant (685), followed by light response elements (609) and abscisic acid response elements (289) (Figure S2, Table S3). Furthermore, several CAREs in the promoter regions were linked to anaerobic induction (ARE), meristem expression (CAT-box), hypoxia-specific induction (GC-motif), enhancer binding protein (CCAAT-box), and other related elements (Figure 5, Table S3). Overall, these results provided valuable insights into the intricate regulatory mechanisms of the *CgCBM* gene family in various developmental, pathogenic, and stress-related processes.

### 2.6. Gene Ontology (GO) Enrichment Analysis

**Figure 6:**
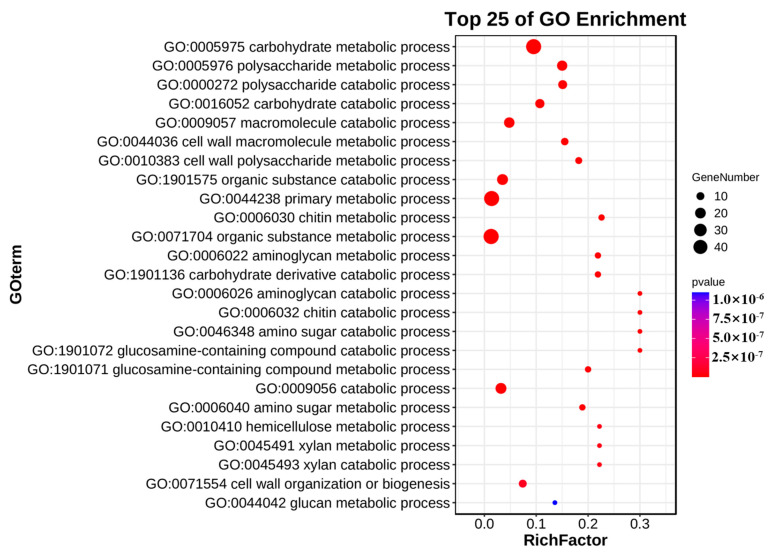
Gene ontology enrichment analysis of *CgCBM* genes.

GO enrichment analysis is a method to compare protein sequences with protein functions of known species to help researchers understand gene functions. In this study, we conducted GO analysis on the *CgCBM* gene family (Figure 6, Table S4). The results revealed that *CgCBM* genes were significantly enriched in various GO terms, including the carbohydrate metabolic process (GO: 0005975), primary metabolic process (GO: 0044238), organic substance metabolic process (GO: 0071704), organic substance catabolic process (GO: 1901575), and macromolecule catabolic process (GO: 0009057). These findings suggest that *CgCBM*s may be involved in influencing metabolic processes, especially the catabolism of carbohydrates and other macromolecular organic compounds.

### 2.7. CgCBM Gene Family Response During C. graminicola Infection

**Figure 7:**
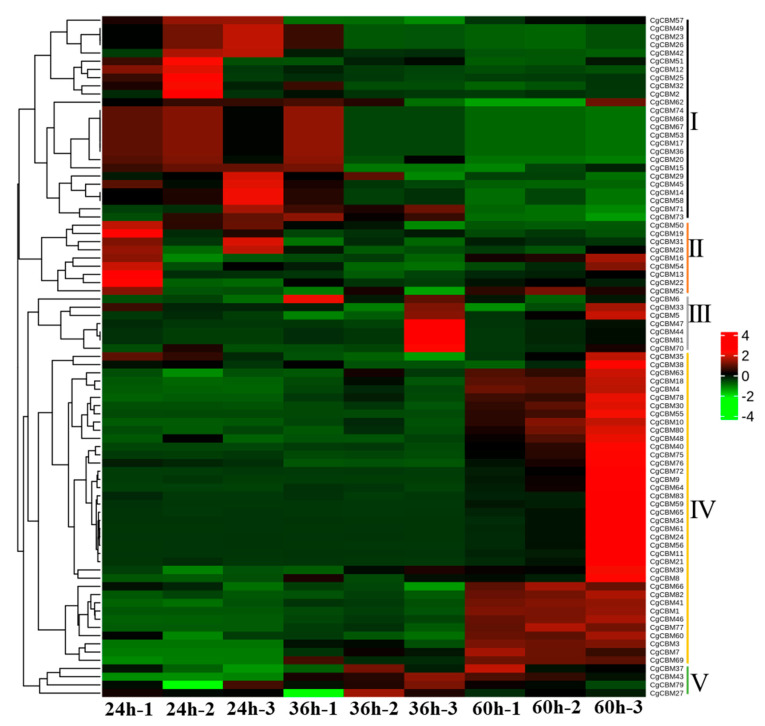
Expression level of *CgCBMs* based on RNA-seq data. Note: Different colors represent different FPKM values. Red and blue indicate high and low expression levels of *CgCBMs*, respectively.

**Figure 8:**
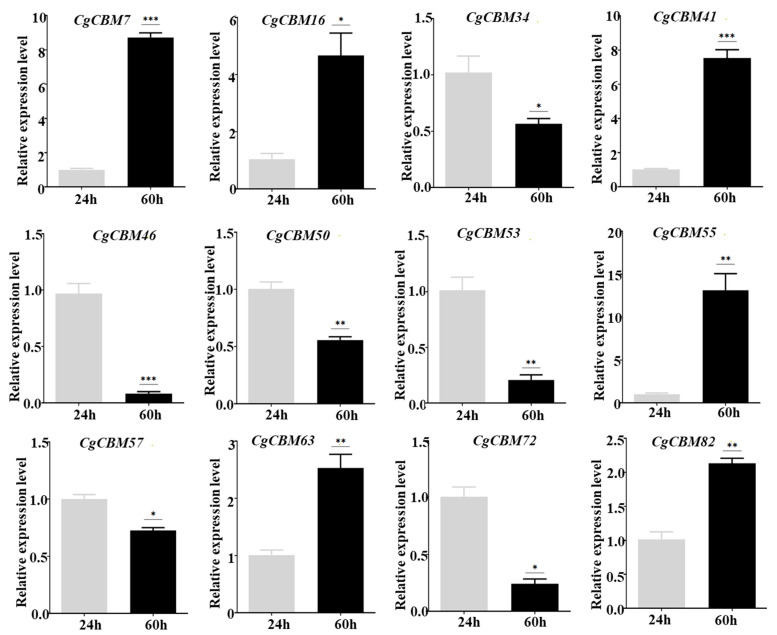
RT-qPCR verification of *CgCBM* expression pattern. Note: Bars represent mean values of three technical replicates ± SE, Student’s *t*-test (*n* = 3, * *p* < 0.05, ** *p* < 0.01, *** *p* < 0.001).

In order to clarify the pathogenic mechanism of *CgCBM* gene family members, the expression levels of the 83 *CgCBM* genes during infection were analyzed using RNA-seq data [26]. Based on the FPKM values of these genes, a heatmap of the expression profiles at three time points post-inoculation (24 h, 36 h, and 60 h) was constructed (Figure 7). The results reveal that the changes in the 83 *CgCBM* gene family members could be categorized into five distinct groups according to the changes observed at different time points. Class I comprised 25 genes, which were gradually down-regulated during infection, while Class IV included 38 genes, which were significantly up-regulated after 60 h of inoculation. It is worth noting that nine genes in Class II were initially down-regulated during infection and then up-regulated. There were seven genes in Class III, and four genes in Class V that had low expression levels at 24 h after inoculation with *C. graminicola* and were moderately up-regulated at 36 h and 60 h. Twelve *CgCBM* genes from different classes were selected for RT-qPCR verification (Table S5). RT-qPCR results confirmed the accuracy of transcriptome data (Figure 8). These *CgCBM* gene family members exhibited significant changes in expression patterns during pathogen infection, indicating their potentially pivotal role in pathogenicity.

## 3. Discussion

Carbohydrates play an essential role in numerous biological functions, ranging from metabolism to energy storage, and provide structural support. CBMs typically function as auxiliary structural domains that can fold independently and accurately recognize a wide array of carbohydrate structures [35]. These versatile CBMs can be present across different biological domains, particularly in proteins that identify polysaccharides [10,11,12]. Their primary function involves the specific recognition and binding of carbohydrates, which leads to various biological effects, including enhanced hydrolysis of insoluble substrates, proximity of the catalytic domain to the substrate, alteration of polysaccharide structures, anchoring of cell surface proteins, and other essential functions [14,15,16].

In this study, 83 genes of the *CBM* family in *C. graminicola* TZ-3 were identified, and their sequences were analyzed by bioinformatics. Previous findings suggested that only 11 *CBM* genes were identified in a pathogenic fungus isolated from rice [36]. The disparity in the number of *CBM* genes among different fungi could be attributed to the varying sizes of fungal genomes. An analysis of structural domains revealed the diverse domain compositions of CgCBMs, indicating their potential multifunctionality. For instance, CgCBM25 comprises five domains, while CgCBM2, CgCBM41, and CgCBM43 each possess four domains that are likely to be functionally interrelated and coordinate. Interestingly, some CgCBMs contain both GH and CBM domains. For example, CgCBM39 contains the CBM6-CBM35-CBM36_like superfamily and the Glyco_hydro_26 superfamily, and CgCBM65 includes the CBM_1 superfamily and the Glyco_hydro_6 superfamily. Earlier research has demonstrated that the phytopathogenic fungus modulates plant immunity through interactions between the GH12 protein and CBM1 [37]. The CBM1 domain inhibits GH12-induced cell death, enabling the pathogen to manipulate plant immunity by synergistically utilizing GH12 and CBM1 proteins. The distinctive structures of CgCBM39 and CgCBM65 may also allow them to assume a specific role in the regulation of plant immunity. These interconnected domains within CgCBMs may function collaboratively during the interactions between pathogens and their hosts.

The phylogenetic analysis of the 83 CgCBM family proteins reveals obvious clustering, and CgCBMs with similar domains tended to be clustered together. CgCBMs found on distinct branches indicate more remote evolutionary connections, implying potential disparities in both structure and function. This analysis unveils the evolutionary lineage and background of these proteins. The varying counts of introns play a critical role in gene function during evolution. Genes with fewer introns may respond more quickly to environmental pressures [38,39,40]. In numerous microorganisms, the distribution of exons and introns in the genes coding for proteins reflects discrete patterns [41]. In this study, CgCBM3 and CgCBM37 contain up to 14 introns, while several other *CgCBM* genes lack introns, indicating potential losses and gains of exons and introns during evolution. Predictions concerning the subcellular localization of CgCBM proteins indicate their primary extracellular localization, suggesting a role in secretion or transport across the cell membrane.

CAREs play a crucial role in determining gene function as they interact with specific transcription factors located upstream of the genes. Their presence in the promoter regions significantly impacts gene function and regulation [42]. Previous studies have found a variety of CAREs related to developments, defense mechanisms, plant hormones, and stress responses were identified in the promoter regions of *CgCBMs*. Our analysis revealed many CAREs involved in light responsiveness, including G-box, Sp1, and GT1 motifs, as well as CAREs related to growth, such as O2-site and CAT-box. In addition, we found CAREs associated with plant hormones, with research indicating that ABREs are linked to responses to abiotic stress [43,44]. Additionally, we detected CAREs involved in multi-factor stress responses, including LTR, ARE, and GC-motifs, which are commonly involved in cold response, physical defense, and stress adaptation. Several studies have highlighted the critical role of CAREs [45,46,47]. However, the exploration and report of CAREs in fungi remain limited. Exploring fungal regulatory elements is vital for revealing genes that contribute to fungal adaptation and pathogenicity.

The GO analysis of the *CgCBM* genes indicated that they mainly affected primary metabolic processes and organic compound metabolic processes. They especially played an important role in the processes of cell wall polysaccharide and amino polysaccharide metabolism, both of which are vital for cell growth, morphological maintenance, and environmental adaptation [48,49,50]. Polysaccharides found in the cell wall, including cellulose, chitin, and pectin, are vital for providing structural support and protection to the cell. The processes involved in the synthesis, degradation, and modification of these polysaccharides are critical for cell growth and division and also affect the ability of cells to respond to environmental stress. Amino polysaccharides, such as glucans and chitosans, are involved in the resistance and mechanical support functions of the cell wall, playing an important role in shaping cellular morphology and maintaining cell wall strength under adverse conditions. These analysis results suggest that *CgCBM* genes may be related to the pathogenic mechanisms of pathogens, which could contribute to the establishment of fungal infections.

The characteristics of gene expression are generally related to their functions [51,52,53]. The *CgCBM* genes may play an important role in both initiating fungal infections and expanding lesions. In this study, we also investigated the expression patterns of the *CgCBM* genes throughout the pathogenic process. At different stages of pathogen infection, the differential expression levels of the *CgCBM* genes vary, indicating that they are involved in the interaction between the pathogen and its host. Some *CgCBM* genes are closely linked to the early phases of fungal infection, with their transcription levels gradually decreasing as the infection progresses. Conversely, the transcription levels of certain genes increase over time, which seems to contribute to the expansion of the lesion. Additionally, certain genes may function in the middle of fungal infections. As the infection progresses, the transcription levels initially increase and then decrease. These findings emphasize the different roles of *CgCBM* genes at various time points during infection, demonstrating their functional diversity.

The findings presented here have laid the groundwork for a comprehensive understanding of the *CgCBM* genes. Through whole-genome analysis, we managed to make preliminary characterizations of these *CgCBMs*. However, additional research is necessary to unravel the functions and molecular mechanisms associated with the *CgCBM* genes. Specifically, the downstream reactions of these genes after being expressed in *C. graminicola* remain unclear. It is of crucial importance to identify the host-triggered genes that interact with *CgCBMs*, which will offer deeper insights into their roles and regulatory pathways.

## 4. Materials and Methods

### 4.1. Identification of CgCBM Gene Family

The *CBM* gene family in *C. graminicola* TZ-3 was identified by retrieving genomic data from previously reported study [34]. The CAZy database (http://www.cazy.org/, accessed on 2 October 2024) was utilized to identify CMB proteins within the genome of *C. graminicola*, using an e-value threshold of less than 1 × 10^−5^. The ExPASy online tools available at https://web.expasy.org/protparam/ (accessed on 4 October 2024) and the PSORT tools available at https://wolfpsort.hgc.jp/ (accessed on 4 October 2024) were utilized to predict the properties of the CgCBM proteins [54].

### 4.2. Multiple Sequence Alignment and Phylogenetic Analysis

Multiple sequence alignment was performed using the Clustal W program. Subsequently, the phylogenetic tree analyses of the sequences were performed using the neighbor-joining method of MEGA 5.0 with a bootstrap value of 1000 replicates. Furthermore, refinement of the phylogenetic tree was achieved using the EVOLVIEW website (http://evolgenius.info//evolview-v2/, accessed on 28 October 2024) [55].

### 4.3. Gene Structure, Protein Motifs, and Protein Structure Analysis

The Gene Structure Display Server was employed to predict gene structures [56]. MEME was used to identify conserved motifs (pattern count = 10, default parameters) [57]. The secondary structures of the CgCBM proteins were predicted using the online tool available at https://npsa.lyon.inserm.fr/cgi-bin/npsa_automat.pl?page=/NPSA/npsa_sopma.html (accessed on 28 December 2024). The three-dimensional structures of the CgCBMs were generated using the online resource available at https://www.swissmodel.expasy.org/ (accessed on 28 December 2024) [58].

### 4.4. Promoter CARE Identification and GO Analysis

The 2000 bp upstream promoter sequences of the *CgCBM* genes were retrieved from the *C. graminicola* TZ-3 genome [34]. The PlantCARE online tool (http://bioinformatics.psb.ugent.be/webtools/plantcare/html/, accessed on 6 October 2024) was employed for CARE identification, while GO functional enrichment analysis was conducted using the website (https://www.omicshare.com/tools, accessed on 6 October 2024) [59,60].

### 4.5. Analysis of CgCBM Gene Expression Patterns

The gene expression patterns of the *CgCBM* genes at different infection time points were investigated by collecting RNA-seq data of *C. graminicola* from the literature [26]. A heatmap based on FPKM values was generated through the online resource (https://www.omicshare.com/tools, accessed on 6 October 2024) [60].

### 4.6. RT-qPCR Validation

Primer Premier 5.0 was utilized for primer design. Total RNA was extracted from infected leaves using the Tiangen DP441 RNA extraction kit (Tiangen, Beijing, China). Following this, cDNA synthesis was conducted using the HiScript III 1st Strand cDNA Synthesis Kit (Vazyme, Nanjing, China). Additionally, the Taq Pro Universal SYBR qPCR Master Mix kit (Vazyme, Nanjing, China) was used to set up the reaction system. Quantitative analysis was performed with the ABI 7500 real-time system, and gene expression levels were assessed using the 2^−∆∆CT^ method, with *UBQ* serving as the internal reference gene.

## 5. Conclusions

In this study, we conducted a comprehensive bioinformatics analysis of the *CgCBM* gene family, emphasizing the expression patterns of *CgCBM* genes to deepen our understanding of this genetic family. We identified a total of 83 CgCBM proteins from the genome of *C. graminicola* and elucidated their physicochemical properties and potential biological functions. This study establishes a theoretical framework for investigating the functions of *CgCBM* genes and the underlying mechanisms of pathogenicity, thereby paving the way for future research on the functional roles of fungal *CBM* gene families.

## Figures and Tables

**Table 1:** The characteristics of carbohydrate-binding modules (CBMs) in *Colletotrichum graminicola* that were detected.

Proposed Gene Name	Gene ID	Superfamily	CDS Length (bp)	Protein Length (aa)	Mw (KDa)	PI	GRAVY	Predicted Subcellular Localization
*CgCBM1*	EVM0000082	CBM24	963	320	33,382.91	5.06	−0.266	extracellular, including cell wall
*CgCBM2*	EVM0000127	CBM18	2001	666	71,878.92	5.35	0.046	extracellular, including cell wall
*CgCBM3*	EVM0000499	CBM18	5337	1778	198,296.45	5.04	−0.487	extracellular, including cell wall
*CgCBM4*	EVM0000502	CBM43	1542	513	55,462.53	4.52	−0.301	extracellular, including cell wall
*CgCBM5*	EVM0000503	CBM1	1224	407	43,312.29	4.97	−0.13	extracellular, including cell wall
*CgCBM6*	EVM0000538	CBM18	921	306	32,464.26	4.96	−0.255	extracellular, including cell wall
*CgCBM7*	EVM0000611	CBM1	951	316	32,771.82	8.81	−0.211	extracellular, including cell wall
*CgCBM8*	EVM0000629	CBM6	1389	462	49,678.22	5.77	−0.176	extracellular, including cell wall
*CgCBM9*	EVM0000654	CBM1	1161	386	40,615.26	5.74	−0.103	extracellular, including cell wall
*CgCBM10*	EVM0001000	CBM35	1653	5 50	59,829.09	4.88	−0.167	extracellular, including cell wall
*CgCBM11*	EVM0001020	CBM1	1620	539	56,672.52	5.24	−0.365	extracellular, including cell wall
*CgCBM12*	EVM0001035	CBM18	1449	482	52,994.38	4.93	−0.295	extracellular, including cell wall
*CgCBM13*	EVM0001094	CBM18	1383	460	47,998.9	4.57	−0.333	extracellular, including cell wall
*CgCBM14*	EVM0001221	CBM50	1077	358	38,031.09	6.2	−0.021	extracellular, including cell wall
*CgCBM15*	EVM0001353	CBM20	1149	382	40,654.17	5.65	−0.17	extracellular, including cell wall
*CgCBM16*	EVM0001383	CBM20	921	306	33,028.77	7.66	−0.831	mitochondrion
*CgCBM17*	EVM0001391	CBM50	777	258	26,733.85	4.67	0.036	extracellular, including cell wall
*CgCBM18*	EVM0001739	CBM48	2121	706	80,373.5	5.81	−0.412	cytosol
*CgCBM19*	EVM0001869	CBM50	2253	750	79,119.78	4.32	−0.114	extracellular, including cell wall
*CgCBM20*	EVM0002259	CBM50	882	293	32,032.8	4.9	−0.43	mitochondrion
*CgCBM21*	EVM0002467	CBM1	1056	351	35,518.81	6.35	−0.026	extracellular, including cell wall
*CgCBM22*	EVM0002471	CBM32	3216	1071	117,653.96	5.18	−0.196	extracellular, including cell wall
*CgCBM23*	EVM0002549	CBM63	960	319	33,342.23	5.83	−0.166	extracellular, including cell wall
*CgCBM24*	EVM0002687	CBM1	852	283	29,396.04	8.69	−0.385	extracellular, including cell wall
*CgCBM25*	EVM0002966	CBM18	1920	639	66,115.14	6.45	−0.29	extracellular, including cell wall
*CgCBM26*	EVM0003407	CBM63	960	319	33,342.23	5.83	−0.166	extracellular, including cell wall
*CgCBM27*	EVM0003421	CBM20	1839	612	66,546.33	4.87	−0.163	extracellular, including cell wall
*CgCBM28*	EVM0003533	CBM35	1500	499	53,921.32	6.17	−0.111	mitochondrion
*CgCBM29*	EVM0003589	CBM18	1176	391	41,894.01	4.85	−0.243	extracellular, including cell wall
*CgCBM30*	EVM0003618	CBM18	1200	399	43,075.59	6.81	−0.179	extracellular, including cell wall
*CgCBM31*	EVM0003780	CBM66	2520	839	88,883.22	4.63	−0.032	extracellular, including cell wall
*CgCBM32*	EVM0003863	CBM24	1077	358	37,630.12	4.87	−0.218	extracellular, including cell wall
*CgCBM33*	EVM0003938	CBM50	2256	751	80,233.22	4.74	−0.154	extracellular, including cell wall
*CgCBM34*	EVM0004131	CBM1	1008	335	33,265.01	6.18	−0.049	extracellular, including cell wall
*CgCBM35*	EVM0004203	CBM20	1941	646	69,141.57	4.61	−0.702	cytosol
*CgCBM36*	EVM0004291	CBM24	3324	1107	122,673.36	6.28	−0.312	extracellular, including cell wall
*CgCBM37*	EVM0004893	CBM18	5196	1731	191,259.37	6.03	−0.409	cytosol
*CgCBM38*	EVM0004906	CBM18	1485	494	51,728.95	6.81	−0.173	extracellular, including cell wall
*CgCBM39*	EVM0004986	CBM35	1419	472	51,672.63	4.64	−0.186	extracellular, including cell wall
*CgCBM40*	EVM0005086	CBM1	870	289	31,117.01	8.01	−0.345	extracellular, including cell wall
*CgCBM41*	EVM0005163	CBM40	3147	1048	113,317.53	4.59	−0.195	extracellular, including cell wall
*CgCBM42*	EVM0005488	CBM50	462	153	15,804.15	4.03	0.477	extracellular, including cell wall
*CgCBM43*	EVM0005686	CBM18	1764	587	59,162.37	5.02	−0.152	extracellular, including cell wall
*CgCBM44*	EVM0005703	CBM24	2538	845	92,835.15	5.27	−0.225	extracellular, including cell wall
*CgCBM45*	EVM0006385	CBM42	1521	506	52,035.03	5.11	−0.132	extracellular, including cell wall
*CgCBM46*	EVM0006399	CBM35	1365	454	49,380.47	4.81	−0.259	extracellular, including cell wall
*CgCBM47*	EVM0006945	CBM24	759	252	26,404.62	4.36	−0.097	extracellular, including cell wall
*CgCBM48*	EVM0007166	CBM18	1233	410	44,380.22	5.93	−0.172	extracellular, including cell wall
*CgCBM49*	EVM0007288	CBM63	960	319	33,342.23	5.83	−0.166	extracellular, including cell wall
*CgCBM50*	EVM0007316	CBM63	1239	412	43,632.58	6.36	0.078	extracellular, including cell wall
*CgCBM51*	EVM0007365	CBM50	1056	351	37,708.2	6.32	−1.097	nucleus
*CgCBM52*	EVM0007452	CBM50	561	186	20,827.2	4.66	−0.462	plasma membrane
*CgCBM53*	EVM0007500	CBM50	426	141	15,667.5	4.23	−0.341	nucleus
*CgCBM54*	EVM0007683	CBM20	1962	653	69,663.82	6.52	−0.066	extracellular, including cell wall
*CgCBM55*	EVM0007697	CBM1	918	305	30,995.61	7.59	−0.057	extracellular, including cell wall
*CgCBM56*	EVM0008001	CBM1	1143	380	40,040.78	4.64	−0.003	extracellular, including cell wall
*CgCBM57*	EVM0008090	CBM21	2289	762	82,729.83	9.73	−0.767	nucleus
*CgCBM58*	EVM0008241	CBM50	1077	358	38,031.09	6.2	−0.021	extracellular, including cell wall
*CgCBM59*	EVM0008309	CBM1	1158	385	41,761.39	5.58	−0.304	extracellular, including cell wall
*CgCBM60*	EVM0008341	CBM24	987	328	33,594.13	5.32	−0.185	extracellular, including cell wall
*CgCBM61*	EVM0008379	CBM1	2643	880	92,236.64	5.63	−0.205	extracellular, including cell wall
*CgCBM62*	EVM0008549	CBM18	3939	1312	143,475.69	5.3	−0.317	extracellular, including cell wall
*CgCBM63*	EVM0009054	CBM48	1728	575	59,481.89	4.54	−0.443	cytosol
*CgCBM64*	EVM0009089	CBM1	978	325	33,476.52	8.27	−0.091	extracellular, including cell wall
*CgCBM65*	EVM0009149	CBM1	1434	477	49,622.7	5.56	−0.047	extracellular, including cell wall
*CgCBM66*	EVM0009225	CBM13	1389	462	46,657.17	4.93	−0.305	extracellular, including cell wall
*CgCBM67*	EVM0009341	CBM50	741	246	26,260.82	4.32	−0.369	cytosol
*CgCBM68*	EVM0009482	CBM50	360	119	12,843.48	5.98	−0.076	cytosol
*CgCBM69*	EVM0009559	CBM50	2130	709	75,858.13	3.94	−0.241	extracellular, including cell wall
*CgCBM70*	EVM0009621	CBM1	1347	448	47,154.66	6.43	−0.234	extracellular, including cell wall
*CgCBM71*	EVM0009624	CBM50	465	154	15,742.17	6.03	0.303	extracellular, including cell wall
*CgCBM72*	EVM0009684	CBM1	1257	418	44,219.35	6.64	−0.142	extracellular, including cell wall
*CgCBM73*	EVM0009752	CBM50	243	80	8180.3	6.69	0.285	extracellular, including cell wall
*CgCBM74*	EVM0009908	CBM24	810	269	30,087.04	8.36	−0.573	mitochondrion
*CgCBM75*	EVM0009927	CBM1	1287	428	46,292.2	5.1	−0.248	extracellular, including cell wall
*CgCBM76*	EVM0010126	CBM1	1128	375	39,811.85	6.29	−0.048	extracellular, including cell wall
*CgCBM77*	EVM0010284	CBM1	936	311	32,713.51	5.31	−0.261	extracellular, including cell wall
*CgCBM78*	EVM0010574	CBM50	2706	901	95,706.82	4.41	−0.085	extracellular, including cell wall
*CgCBM79*	EVM0010641	CBM43	1611	536	56,805.39	4.61	−0.171	extracellular, including cell wall
*CgCBM80*	EVM0010800	CBM18	3363	1120	122,267.1	5.54	−0.391	extracellular, including cell wall
*CgCBM81*	EVM0011047	CBM24	843	280	30,681.4	4.83	−0.29	cytosol
*CgCBM82*	EVM0011272	CBM18	1605	534	56,801.19	5.86	−0.364	extracellular, including cell wall
*CgCBM83*	EVM0011275	CBM1	786	261	27,581.84	6.87	−0.051	extracellular, including cell wall

ID: identity; bp: base pair; aa: amino acids; PI: isoelectric point; Mw: molecular weight; GRAVY: grand average of hydropathicity; KDa: kilo dalton.

## Data Availability

Data is contained within the article and Supplementary Material.

## Supplementary Materials

The following supporting information can be downloaded at: https://www.mdpi.com/article/10.3390/ijms26030919/s1.
