# Supplementary material for: Genome-Wide Identification and Analysis of Carbohydrate-Binding Modules in Colletotrichum graminicola
Source: Int J Mol Sci. 2025 Jan 22;26(3):919. doi: 10.3390/ijms26030919 (PMC11817085; doi:10.3390/ijms26030919)
Supplement: Supplementary file 1 [file ijms-26-00919-s001.zip › Figure S1, S2.pptx]

## Slide 1
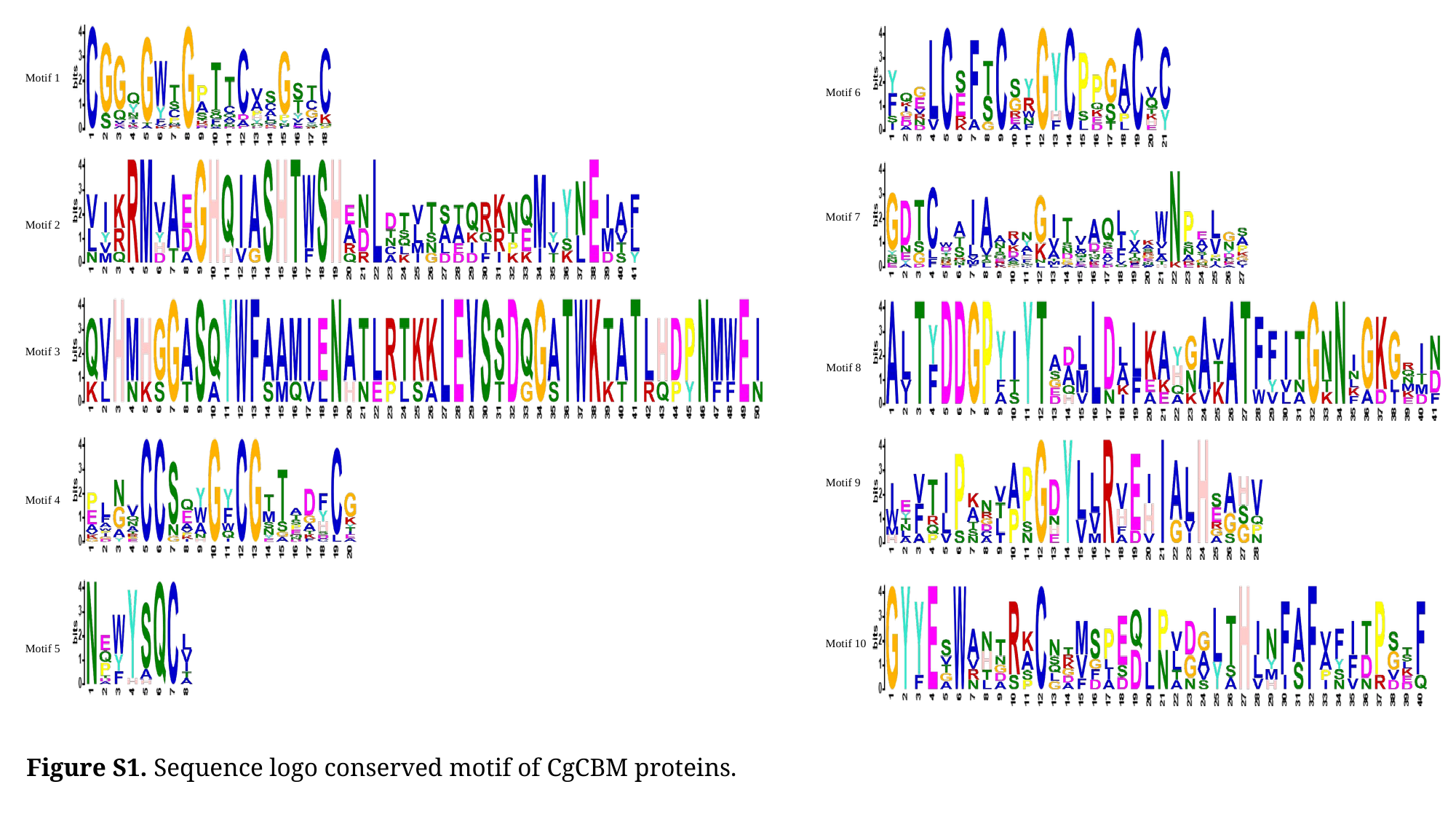

Motif 1
Motif 6
Motif 7
Motif 2
Motif 3
Motif 8
Motif 9
Motif 4
Motif 10
Motif 5
Figure S1. Sequence logo conserved motif of CgCBM proteins.

## Slide 2
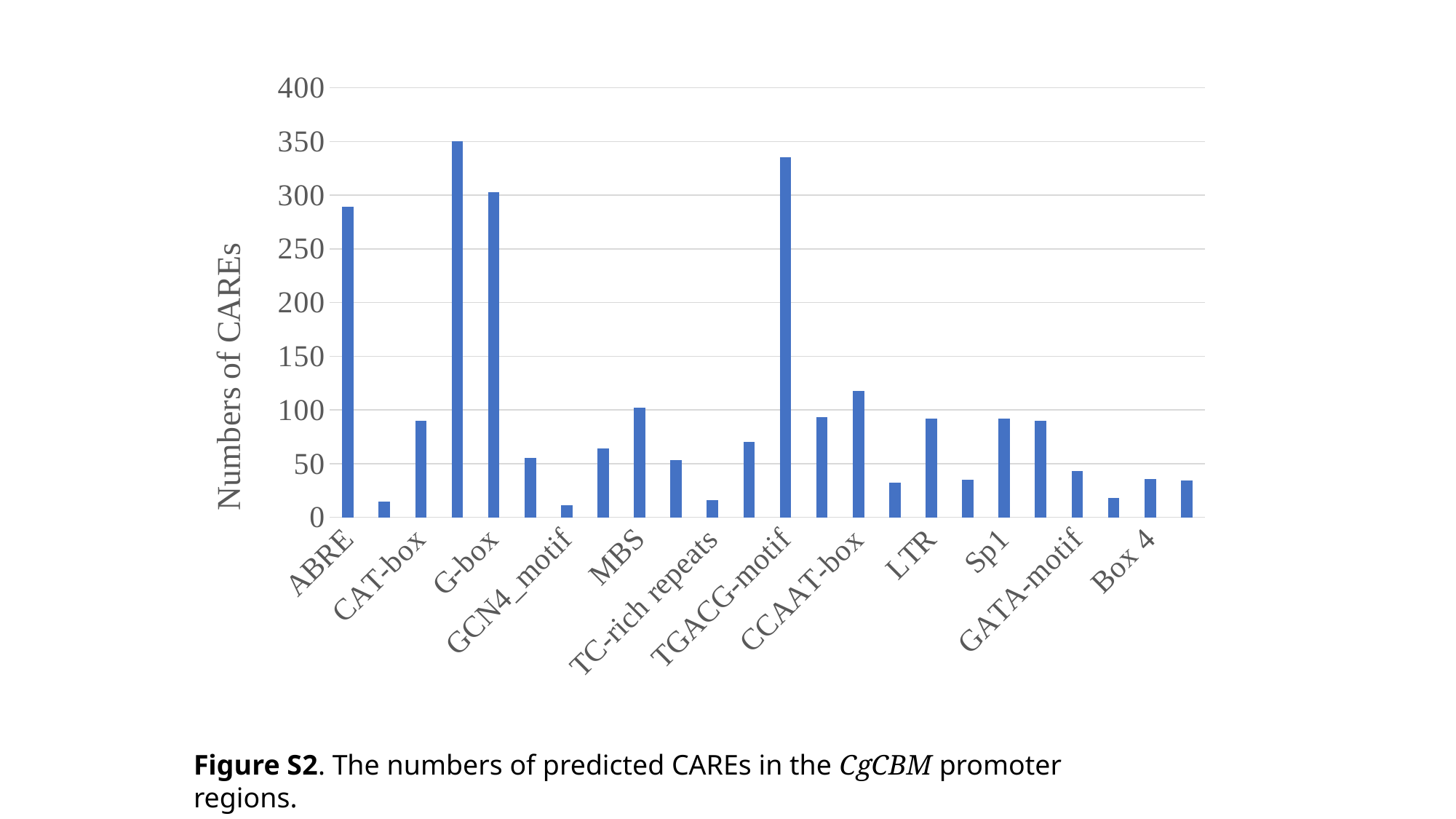

### Chart
| Category | |
|---|---|
| ABRE | 289.0 |
| AuxRR-core | 15.0 |
| CAT-box | 90.0 |
| CGTCA-motif | 350.0 |
| G-box | 303.0 |
| GC-motif | 55.0 |
| GCN4_motif | 11.0 |
| GT1-motif | 64.0 |
| MBS | 102.0 |
| O2-site | 53.0 |
| TC-rich repeats | 16.0 |
| TCT-motif | 70.0 |
| TGACG-motif | 335.0 |
| ARE | 93.0 |
| CCAAT-box | 118.0 |
| GARE-motif | 32.0 |
| LTR | 92.0 |
| P-box | 35.0 |
| Sp1 | 92.0 |
| TGA-element | 90.0 |
| GATA-motif | 43.0 |
| TATC-box | 18.0 |
| Box 4 | 36.0 |
| TCA-element | 34.0 |Figure S2. The numbers of predicted CAREs in the CgCBM promoter regions.
